# Glycerol Immobilises Anaerobic Digestate Supplied Nitrogen

**DOI:** 10.1007/s12649-024-02876-8

**Published:** 2025-01-25

**Authors:** Christina van Midden, Liz Shaw, Jim Harris, Tom Sizmur, Hayden Morgan, Mark Pawlett

**Affiliations:** 1 School of Water, Energy, and Environment, Cranfield University, Bedford, MK43 0AL UK; 2 Department of Geography and Environmental Science, University of Reading, Reading, RG6 6AB UK; 3 Bioprocess Department, Future Biogas Ltd, Guildford, GU2 7YD UK

**Keywords:** Biogas Residue, Glycerol, Nitrogen Immobilisation, Microbial Community, PLFA

## Abstract

Anaerobic digestate, a nutrient rich by-product of the biogas industry, is frequently applied to agricultural land as a fertiliser. However, nitrogen losses from its application negatively impact air and water quality. Therefore, methods are needed to reduce these losses. The aim of this study was to test the efficacy of applying digestate with glycerol, an organic carbon rich by-product of the biodiesel industry, on microbial nitrogen immobilisation and the soil microbial community. Soil was incubated with digestate, applied at a rate equivalent to 250 kg-N ha^-1^, in a laboratory experiment over 50 days with glycerol additions at either 0, 12, 24 or 36 kg-C m^3^ of digestate. The addition of glycerol resulted in significantly higher microbial biomass carbon and increased the relative abundance of Gram-negative bacteria. The 24 and 36 kg-C m^3^ doses of glycerol resulted in similarly greater and longer lasting effect on microbial biomass carbon, indicating that beyond 24 kg-C m^3^ digestate that nitrogen (or other essential nutrients) became the limiting factor for microbial growth instead of carbon. Soil available nitrogen decreased throughout the study and remained at lower concentrations in glycerol treatments than the digestate only treatment by the end of the study. These results demonstrate that glycerol has the potential to reduce nitrogen losses from digestate application by immobilising nitrogen in the microbial biomass. Therefore, the co-application of digestate and glycerol to soil is a potential mechanism for the biogas and biofuel industries to valorise their respective by-products. Further research is needed to verify that this method is viable under field conditions.

## Graphical Abstract

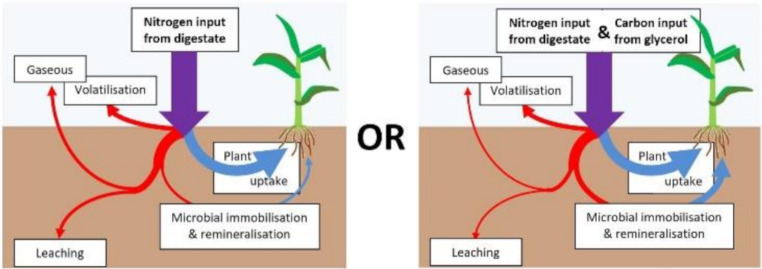

## Introduction

The United Nations Sustainable Development Goals (SDGs) were agreed by 193 Member States in 2015, establishing targets to guide countries to a sustainable future [1]. One of these goals, SDG 7, involves the need for a substantially greater amount of energy to be produced from renewable sources. Energy can be renewably produced from biomass (e.g. biogas, biodiesel), but bioenergy industries create by-products that also require sustainable management, both from an environmental and economic viewpoint.

The anaerobic digestion of organic waste is an expanding industry, producing energy from biologically generated gases. These biogas plants produce a nutrient rich by-product, known as anaerobic digestate, of which over 90% produced in the UK is applied to agricultural land as an alternative to mineral forms of nitrogen [2]. However, there are environmental impacts of digestate application, such as ammonia volatilisation, nitrous oxide (N_2_O) emissions and nitrate leaching [3, 4]. Ammonia volatilisation can account for up to 60% of nitrogen lost from digestate application [5], but the use of low-emission slurry spreading techniques such as acidifying the digestate, or using precision application like bandspreading and injection, have been shown to reduce these losses by 45 to 95% [6–9]. However, there are concerns that these practises could increase nitrate leaching and N_2_O emissions [10–11]. Therefore, it is imperative to find mechanisms to keep the nitrogen supplied by anaerobic digestate within the rooting zone of the soil, where it is beneficial for crop production. As such, these mechanisms need to be used in conjunction with low-emission spreading techniques. Biological immobilisation of nitrogen could provide a sustainable pathway to achieve this objective.

Soil microorganisms immobilise nitrogen within their biomass, a proportion of which upon cell death forms necromass [12]. This immobilised nitrogen is subsequently remineralised when it is primed for decomposition by the release of plant root exudates, which occurs when plants require nitrogen for growth [13]. Therefore, promoting microbial growth could both increase the amount of digestate-supplied nitrogen available to plants by reducing losses and synchronise availability to match plant demand. The growth of most soil microorganisms requires a source of carbon, of which digestate has a low total content of; typically between 0.43 and 3.4% with carbon: nitrogen (C: N) ratios between 1.4 and 6.3 [14]. At these low C: N ratios, microbes are carbon limited and use digestate carbon for metabolism, but digestate supplied nitrogen that is in excess of microbial demand is excreted to soil. Therefore, an additional source of carbon is needed to promote microbial growth and nitrogen immobilisation. Studies on mixing carbon substrates with synthetic nitrogen, which would otherwise supply no carbon to the soil, resulted in positive nitrogen immobilisation [15, 16]. When coating urea with poly-γ-glutamic acid, Xu et al. [17] measured a short-term increase in microbial biomass nitrogen followed by an improved crop yield, evidencing that microbes assisted in ensuring plants were supplied with nitrogen throughout their growing period via nitrogen immobilisation and remineralision.

Glycerol, a by-product of the biodiesel industry, is a labile hydrocarbon which many microorganisms can utilise as a carbon source [18]. Glycerol is created during the transformation process of vegetable oils and fats into biodiesel and makes up around 10% w/w per unit of biodiesel produced [19], resulting in considerable volumes that require disposal. This is restricting the economic growth of the industry as traditional markets for glycerol, which include pharmaceuticals and personal care products, become saturated [20]. Therefore, new ways of utilising glycerol are needed. Several studies have looked at applying glycerol on agricultural soil to immobilise nitrogen, either for reducing environmental pollution [21, 22] or to improve the nitrogen use efficiency of fertilisers [23] by stimulating microbial growth and immobilising nitrogen.

For the co-application of digestate and glycerol to function successfully as a fertiliser, the nitrogen locked within the necromass, resulting from the glycerol stimulated microbial growth, needs to be mineralised by the native soil microbial community into plant available forms. A review on the effects of digestate on the soil microbial community, found that the majority of studies observed no significant difference when compared to synthetic nitrogen addition [24]. There is evidence that microbial utilisation of glycerol produces several intermediate products, such as antimicrobial compounds [25], but how it influences soil microbial community is little researched. Therefore, it is important to understand the potential effects of co-applying digestate and glycerol on soil microbial communities, to ensure its continual provision of nutrient cycling services.

This study aimed to investigate the effects of adding different rates of glycerol into liquid digestate on soil microbial biomass and nitrogen immobilisation. Liquid digestate was selected as it contains higher proportion of nitrogen following separation into solid and liquid fractions [26], a procedure which biogas companies often do due to storage and transport constraints [27]. The objectives were to determine which rate of glycerol would be most effective at inducing nitrogen immobilisation and what impact it would have on the soil microbial community. It was hypothesised that increasing glycerol rates would result in higher levels of microbial nitrogen immobilisation by increasing the availability of carbon to microorganisms. It was also hypothesised that the microbial community composition would change due to the addition of glycerol to digestate. A pot experiment was established to test these hypotheses in the absence of confounding environmental variables, such as temperature and moisture content.

## Method

### Soil, Digestate and Glycerol

A sandy loam topsoil (69% sand, 20% silt, 11% clay) bought from Bourne Amenity Ltd, was used for the study. Liquid digestate was supplied by Future Biogas Ltd from a biogas plant using a mixed feedstock of 85 tonne maize silage, 7.5 tonne cow manure and 18 tonne chicken manure. The plant is mesophilic operating at 43 °C with a retention time of 98 days. Post digestion, the digestate is pasteurised at 70°C for 1 hour. The liquid fraction was collected fresh after separation from the whole digestate by a screw press. Glycerol comprising of 39.2% carbon was bought from Sigma Aldrich. Details on material properties are in Table 1.

**Table 1:** Characterisation of the materials used in the incubation

Properties	Soil	Liquid digestate	Glycerol
Dry matter (%)	84.5	4.8	-
pH	8.0	8.1	-
Total Carbon (g kg^-1^)	32.4	35.1	391.9
Nitrogen			
*Total (g kg*^*-1*^)	2.8	5.1	-
*Ammonium (mg kg*^*-1*^)	0.9	3234	-
*Nitrate (mg kg*^*-1*^)	264	< 10	-
Phosphorous			
*Total (mg kg*^*-1*^)	-	316	-
*Available (mg l*^*-1*^)	49	-	-
Potassium			
*Total (mg kg*^*-1*^)	-	4360	-
*Available (mg l*^*-1*^)	755	-	-
C: N ratio	11.57	3.27	-
Microbial biomass C (mg-C kg^-1^)	251	-	-
Microbial Biomass N (mg-N kg^-1^)	45	-	-

### Soil Incubations

Soil was air dried and sieved to 2 mm to remove any stones and large debris. 150 g (dry weight basis) of soil was added to 330 ml plastic containers (top diameter 8 cm, bottom diameter 5 cm, height 12 cm) without drainage holes. The soil water holding capacity was determined using a saturate and drain method modified from Harding and Ross [28]. Potted soil was then adjusted to 40% water holding capacity, which is optimal for microbial development [29]. The pots were pre-incubated at 20 ± 4 °C in the dark under aerobic conditions for two weeks, to allow soil microbial population to acclimatise after being disturbed and rewetted.

The incubation experiment consisted of five treatments, arranged in a randomised block design with five replications: (1) soil only control (CONT); (2) liquid digestate only (LD); (3) liquid digestate with glycerol at 3% v/v (LD + 3%G); (4) liquid digestate with glycerol at 6% v/v (LD + 6%G); and (5) liquid digestate with glycerol at 9% v/v (LD + 9%G). Five sets were set-up to allow for destructive sampling on five occasions: 3  hours after application, then 7, 14, 30, and 50 days after application. This gave a total of 125 experimental units (5 treatments x 5 replicates x 5 sampling dates).

Digestate was applied at 14 ml per pot, a rate equivalent to 250 kg-N ha^-1^. Before application, the digestate receiving glycerol was mixed with 0.42 ml, 0.84 ml or 1.26 ml glycerol to make mixes of 3%, 6% and 9% glycerol to digestate volume to volume (v/v), adding an extra 12.4, 24.8 and 37.2 kg-C per m^-3^ of digestate, equivalent to 614, 1228 and 1842 kg-C ha^-1^. Water was added to treatments 1–4 to ensure they all received the same total amount of liquid as treatment 5. The amendments were then mixed into the soil. Pots were loosely covered to reduce moisture losses and incubated in the dark at 20 ± 4 °C until sampled. To maintain water holding capacity at 40%, the pots were weighed twice weekly to check moisture content and deionised water was added as required.

### Microbial Analysis

Microbial biomass carbon and nitrogen were determined following the fumigation-extraction method [30]. Two weighed subsamples were taken from each soil sample, one subsample was fumigated for 24 h at 20 °C with CHCl_3_, then extracted with 50 ml of a 0.5 M  K_2_SO_4_ solution and filtered. The second portion was equally processed, but without the CHCl_3_ fumigation step. The organic carbon and nitrogen were determined with an automatic analyser for liquid samples (Shimadzu TOC-V with a TN module). Microbial biomass carbon and nitrogen were calculated as the difference between the carbon and nitrogenextracted from the fumigated samples and those extracted from the non-fumigated samples, multiplied using K_EC_ and K_EN_ values of 0.45 and 0.54 respectively [30, 31].

Phospholipid fatty acid analysis (PLFA) was used to determine the phenotypic structure of the microbial community, based on the method modified from [32] at 3 timepoints: 3 hours, then at days 14 and 50 from application. Microbial lipids were extracted using a Bligh & Dyer solvent (1:2:0.8 (v/v/v) chloroform: methanol: citrate buffer). Lipids were then fractionated using solid phase extraction cartridges (SPE), the polar lipids then methylated and resulting fatty acid methyl esters (FAMES) extracted. The FAMES were analysed by gas chromatography (6890 N Agilent Technologies) following the same procedure as [33]. The relative abundance (%mol) of all PLFAs present in the sample, including the non-specified ones, were used for the analysis of the PLFA patterns. Bioindicator fatty acids used to identify microbial groups were: the sum of i15:0, ai15:0, i16:0, i17:0, ai17:0, 10me18:0 for Gram-positive (G+) bacteria [34, 35], the sum of 16:1ω7c, 17:1ω7, 18:1ω7t, 18:1ω13 for Gram-negative (G-) bacteria [34, 35], 18:2ω6,9 for ectomycorrhizal and saprophytic fungi [34]. Total bacteria were calculated as the sum of G + and G- bacteria. The fungi: bacteria (F: B) ratio was calculated as the fungal biomarker 18:2ω6,9 divided by the sum of the bacterial biomarkers [34].

### Chemical Analyses

Total soil available nitrogen as the sum of ammonia (NH_4_-N) and total oxides of nitrogen (TON-N), which is the sum of nitrite and nitrate, was determined using the potassium chloride extraction method [36]. 20 g of soil was eluted with 100 ml of 2 M KCl solution, filtered and stored at -20 °C until analysed on an analytical segmented flow multi-chemistry analyser (Seal, AA3). Soil pH was measured based on the British Standard Institution method [37]. 10 ml of air-dried soil was mixed with 50 ml of 1 M KCl solution and pH was measured using a Jenway 3520 pH meter.

### Statistics

Statistical analysis was carried out in Statistica version 14. Data was first tested for normality and homoscedasticity. The soil available nitrogen and microbial biomass carbon and nitrogen datasets failed to meet the assumptions for equal variance and were transformed using the Box-Cox function. The differences between treatments were analysed by a one-way analysis of variance (ANOVA) to determine treatment effects at each time point. Significant differences between glycerol rates were determined by Tukey’s post hoc test. All differences were considered statistically significant if *p* < 0.05. Principal Component Analysis was run on the PLFA profile data, which was normalised by measuring each biomarker as the relative abundance (%mol) to all the biomarkers.

## Results

### Impact of Digestate and Glycerol Application on Microbial Growth

**Fig. 1:**
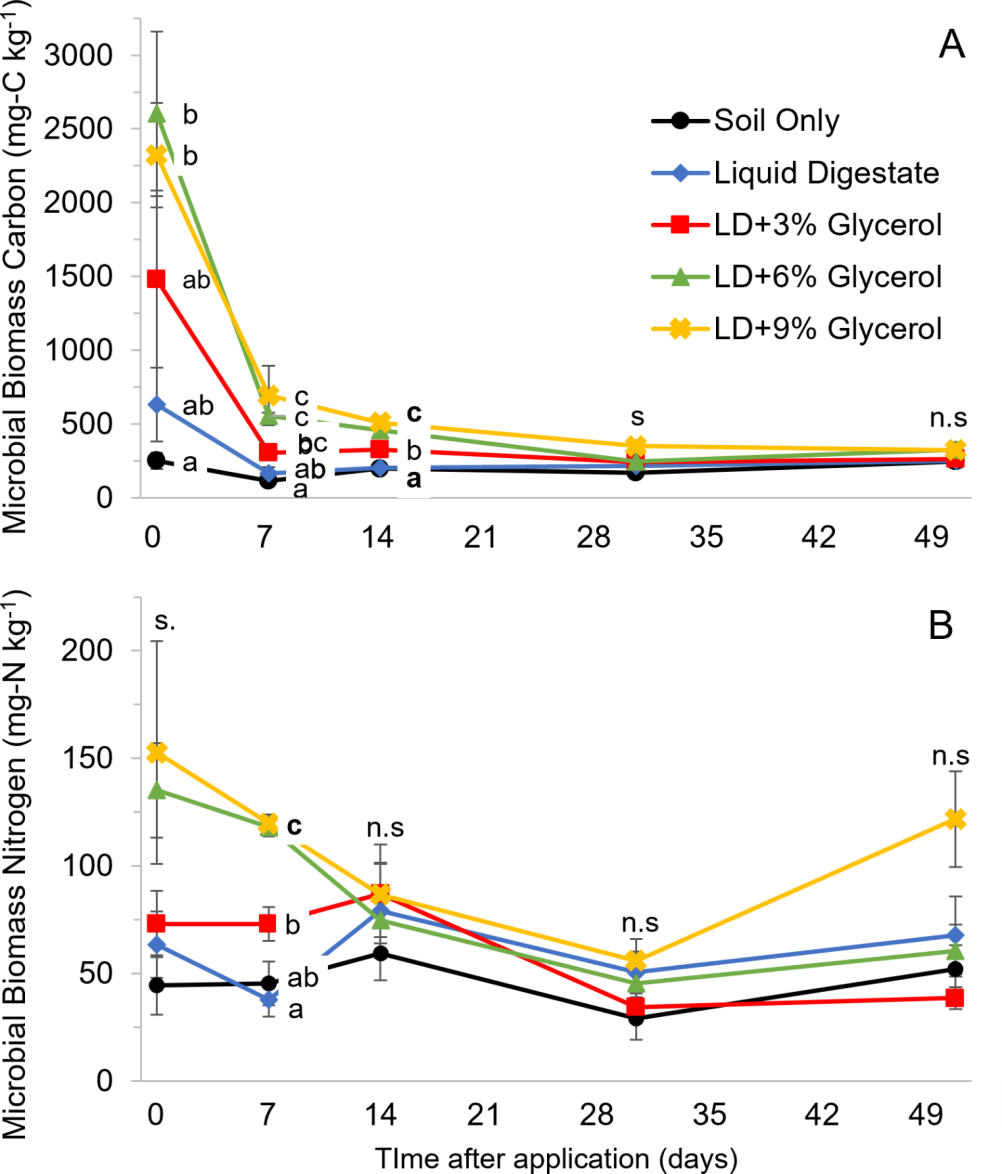
Changes in soil microbial biomass carbon (**A**) and nitrogen (**B**) during the incubation. Points denoting different lowercase letter have statistically different treatments effects according to Tukey’s test at 5% probability. Error bars denote the standard error of the mean, *n* = 5. s = significant at *p* < 0.05, n.s = non-significant at *p* ≥ 0.05

The addition of only digestate to soil (without glycerol) did not result in significantly higher microbial biomass carbon (C) at any timepoint (Fig. 1A). Microbial biomass C was generally higher with increasing glycerol rates, with significantly highest concentrations found at days 7 and 14 under the LD + 6%G and LD + 9%G treatments. At 30 days, only LD + 9%G had a significantly higher biomass C than digestate alone.

### Impact of Digestate and Glycerol Application Nitrogen Dynamics

**Fig. 2:**
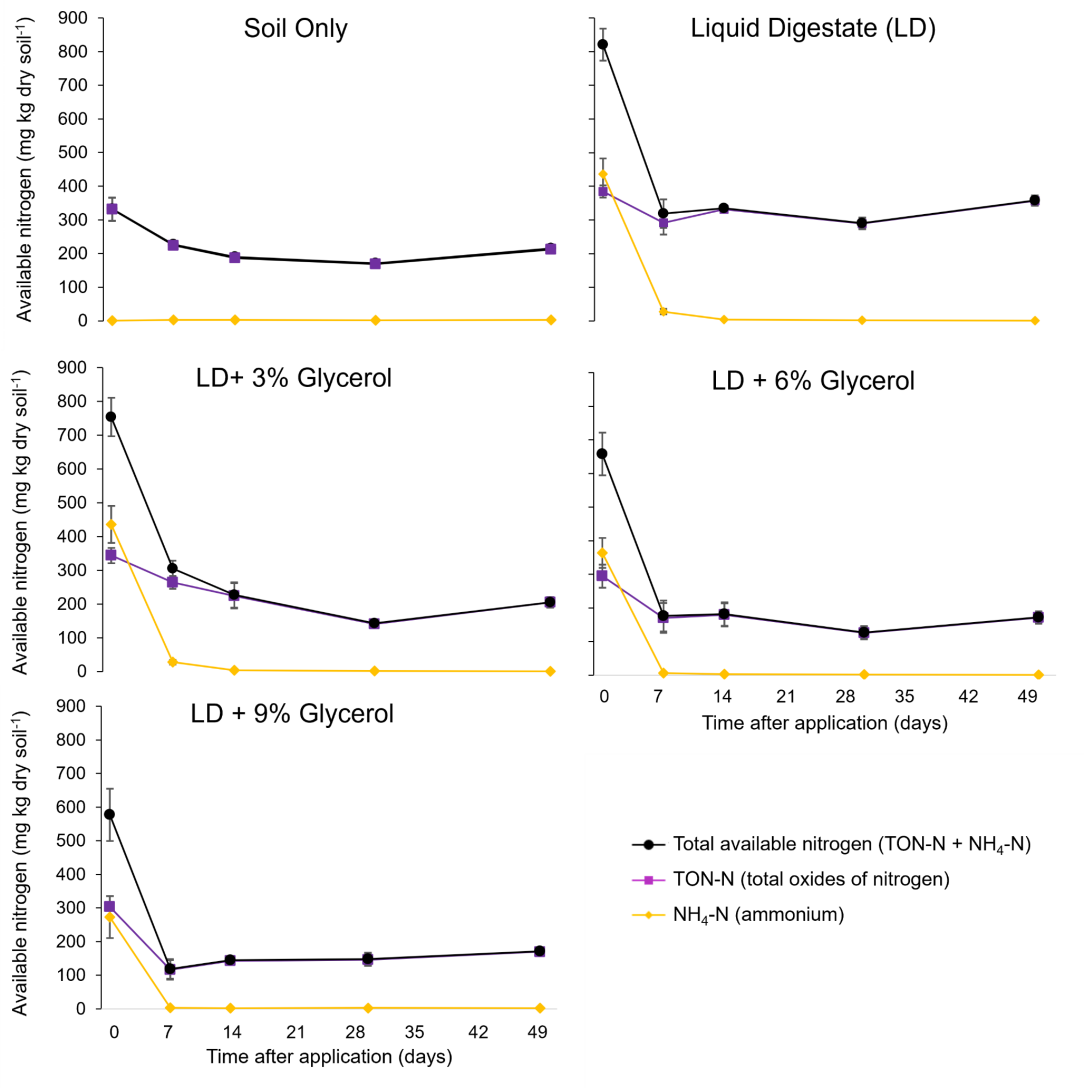
Changes in total soil available nitrogen as the sum of nitrite, nitrate (TON-N) and ammonium (NH_4_-N) during incubation. Error bars denote the standard error of the mean (n=5)

Digestate supplied soil with NH_4_-N, resulting in significantly (*p* < 0.001) higher total soil available nitrogen by 488 mg-N kg^-1^ (Fig. 2) compared to the soil only control 3 hours after application. During the first week of incubation concentrations of NH_4_-N declined by 90%, but total soil available N remained significantly (*p* < 0.002) higher than the soil only treatment for the rest of the incubation (Table 2). From 7 days onwards, the majority of total available N comprised of nitrite and nitrate (Fig. 2). Microbial N was not significantly affected by the addition of digestate to soil at any timepoint (Fig. 1B).

**Table 2:** Changes in mean (± SE) of total soil available nitrogen as sum of ammonium, nitrite and nitrate following treatment incorporations. Mean (*n* = 5) values between treatments in a row (sampling time) denoted with a different lower-case letter are statistically different according to Tukey’s test at the 5% probability level

Sampling time after application	Treatment				
	Soil Only	Liquid Digestate (LD)	LD + 3% Glycerol	LD + 6% Glycerol	LD + 9% Glycerol
3 h	332 ± 34^a^	821 ± 47^b^	754 ± 57^b^	658 ± 63^b^	577 ± 77^b^
7 days	227 ± 8^abc^	319 ± 42^a^	305 ± 23^ab^	176 ± 46^bc^	119 ± 29^c^
14 days	189 ± 10^a^	335 ± 9^b^	227 ± 37^a^	182 ± 35^a^	144 ± 11^a^
30 days	171 ± 10^a^	291 ± 17^b^	143 ± 9^a^	127 ± 19^a^	148 ± 18^a^
50 days	215 ± 9^a^	358 ± 15^b^	205 ± 15^a^	172 ± 18^a^	172 ± 7^a^

A week after application, glycerol addition at LD + 6%G and LD + 9%G had significantly (p< 0.001) lower total soil available N of 129 and 186 mg-N kg^-1^(Table. 2) compared to digestate without glycerol. Microbial N under LD + 6%G and LD + 9%G was significantly (p< 0.001) higher than digestate alone by 94 and 97 mg-N kg^-1^ (Fig. 1b) respectively. Whilst LD + 3%G did not significantly reduce total available N compared to digestate alone (*p* > 0.05), it did have a significantly (*p* < 0.05) higher microbial N concentration of 40 mg-N kg^-1^. From day 14 onwards glycerol at all rates consistently had similarly lower total available nitrogen concentrations that was significantly different (*p* < 0.001) compared to digestate alone (Fig. 2). By the end of the 50-day incubation total soil available nitrogen remained lower than the digestate control by 43–52%.

### Impact of Glycerol and Digestate Application on Soil Microbial Community

**Fig. 3:**
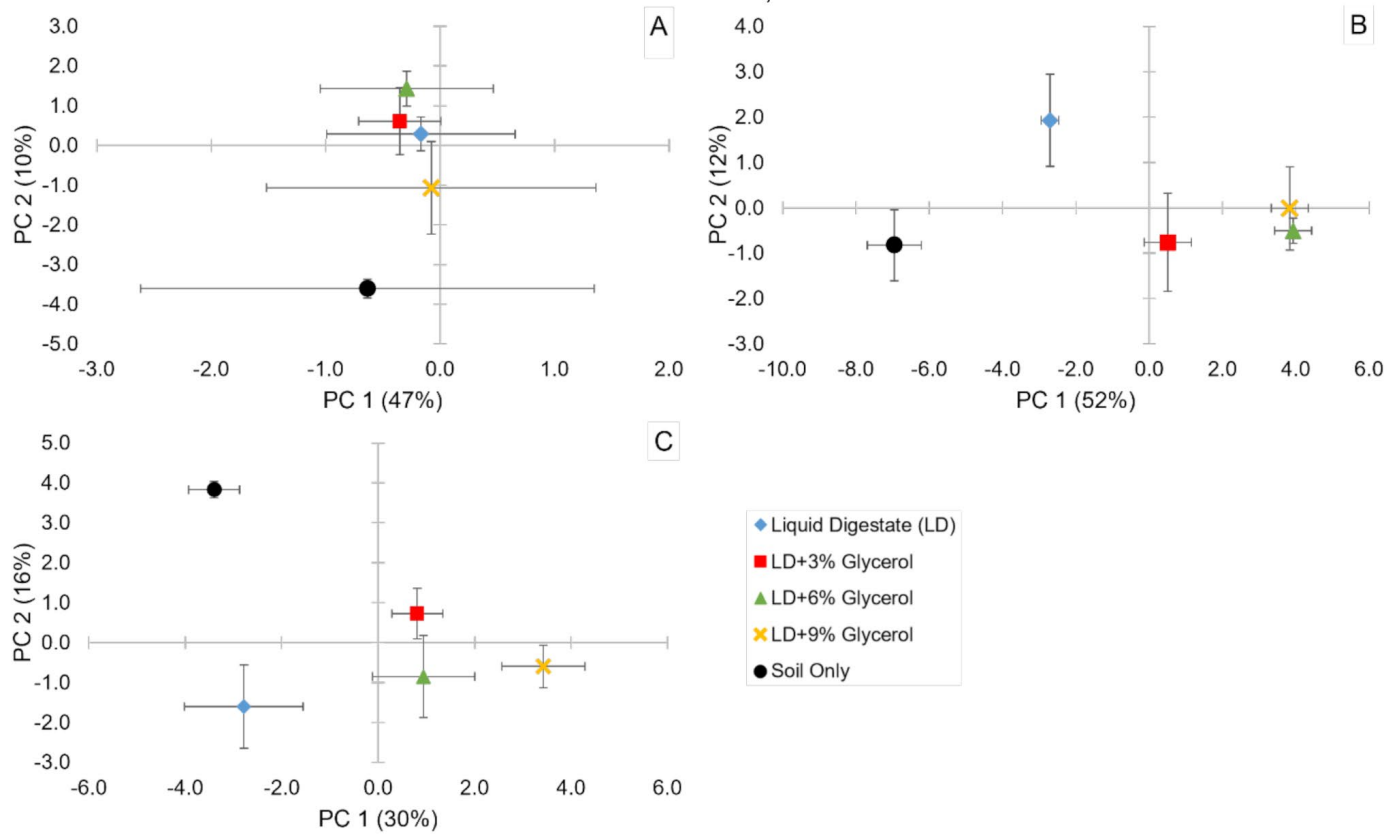
Treatment response of the microbial community identified by Principal Component Analysis of the PLFA data (PC mean ± SE, *n* = 5) at 3 hours (**A**), 14 days (**B**) and 50 days (**C**) after application

The first two principal components (PC) of the PLFA data accounted for over 50% of the total variation at each timepoint (Fig. 3), with significant (*p* < 0.01) effects on the PC2 axis 3 hours after application, on the PC1 axis at day 14 and both PC axes at 50 days. 3 hours after application the unamended control was distinctly separate from a cluster formed by the LD + 0–6%G whilst the LD + 9%G lay between the two. Fatty acid loadings (≥ 0.8 and ≤-0.8) that contributed the most were 16:1ω11t, 16:1ω5, 16:0, Me17:0 isomer1, Me17:0 isomer2, 17:0c, 18:1ω9c, 19:1ω6 and 19:0c, Gram-positive bacteria biomarkers 15:0i, 15:0ai, ai17:0 and 18:0 (10Me), and fungi biomarker 18:2ω6,9 on PC1 and Gram-positive bacterial biomarker 16:0i on PC2. At 14 days the unamended and digestate controls were distinctly separate and further separations were clear between two groups of glycerol additions: LD + 0-3G% and LD + 6–9%G. Fatty acid loadings (≥ 0.8 and ≤-0.8) that contributed the most at day 14 were 16:1ω5, Me17:0 isomer2, 17:0c, 17:1ω8t, 17:0 (12Me), 19:0c, 19:1ω6, 20:4 and 20:5ω3, Gram-positive bacterial biomarkers 16:0i and 18:0 (10Me), Gram-negative bacterial biomarkers 16:1ω7c and 18:1ω13 for PC1 and 16:0 on PC2. At day 50 the unamended and digestate controls continued to be distinctly separate, whilst glycerol addition resulted in distinct separations between 0% and a group of LD + 6–9%G whilst LD + 3%G lay between the two. Fatty acid loadings (≥ 0.8 and ≤-0.8) that contributed the most were 16:0, 18:1ω7t, Gram-positive bacterial biomarker 16:0i, and fungal biomarker 18:2ω6,9 on PC 1 and 17:1ω8c on PC 2.

**Fig. 4:**
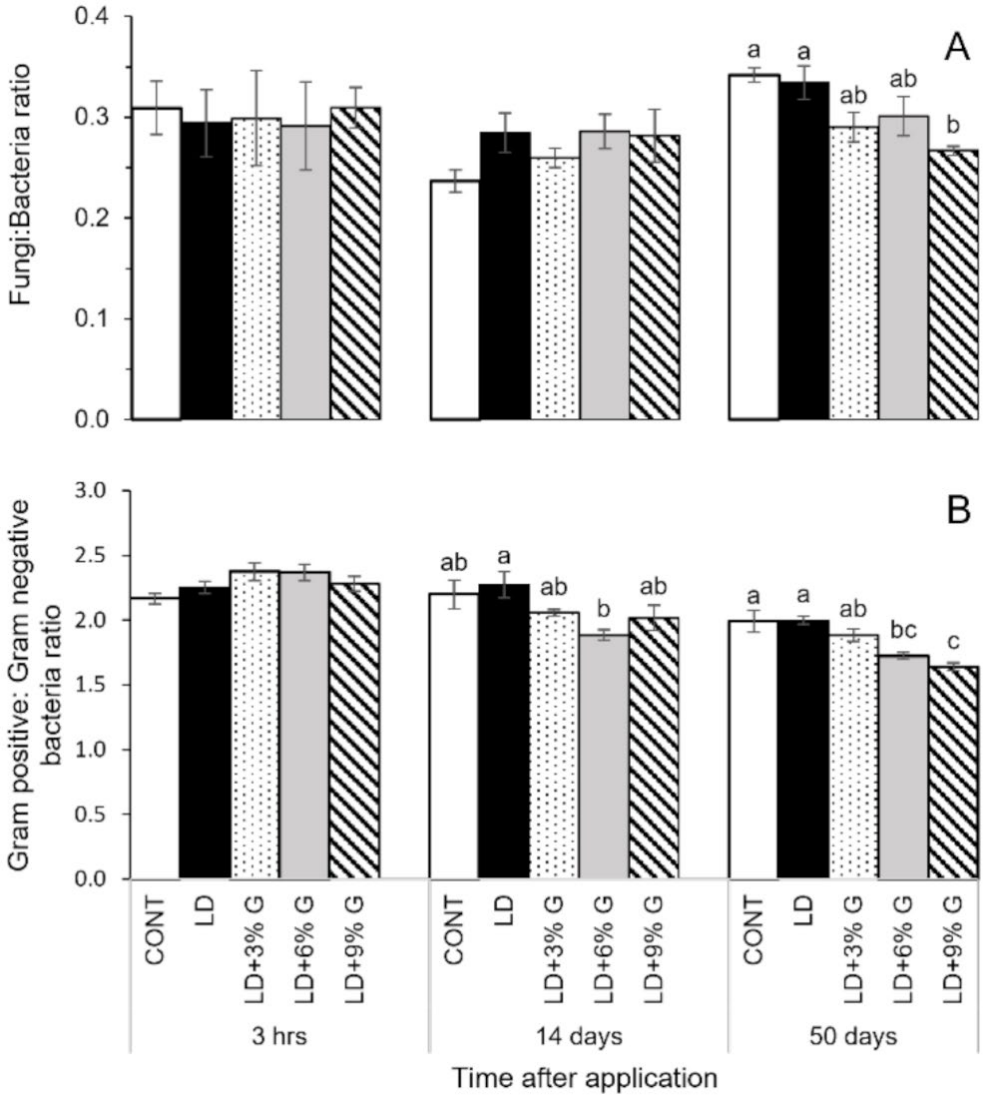
Fungal/Bacteria ratio (**A**) and Gram-positive/Gram-negative ratios (**B**) calculated using PLFA biomarkers on soil samples collected 3 hours, 14 days and 50 days after digestate application. Error bars denote the standard error of the mean (*n* = 5). Cont = soil only; LD = liquid digestate; LD + 3 or 6 or 9%G = liquid digestate + 3 or 6 or 9%v/v glycerol

The addition of only digestate (without glycerol) to soil lead to no significant effects (*p* > 0.05) on the F: B or G+:G- ratios at any timepoint compared to unamended soil (Fig. 4). At day 50, LD + 9%G addition had a lower F: B ratio by 13% than digestate without glycerol. LD + 6%G resulted in a 17% lower G+:G- ratio at day 14 digestate without glycerol. LD + 6–9%G had lower G+:G- ratios of 14% and 18% respectively at day 50 compared to digestate control (Fig. 4B).

### Impact of Digestate and Glycerol Application on Soil pH

Soil pH was higher in treatments with only digestate addition to soil (without glycerol) at 7 days after application, whereafter there was no significant difference (Table 3). The addition of 3% v/v glycerol to digestate resulted in a higher pH at 30 days after application compared to digestate without glycerol. Glycerol addition at 6 and 9% v/v did not result in higher pH values compared to the digestate treatment at any time.

**Table 3:** Changes in soil pH (mean ± SE) following treatment incorporations. Mean (*n* = 5) values between treatments in a row (sampling time) denoted with a different lower-case letter are statistically different according to Tukey’s test at the 5% probability level

Time after application	Treatment				
	Soil only	Liquid Digestate (LD)	LD + Glycerol at 3% v/v	LD + Glycerol at 6% v/v	LD + Glycerol at 9% v/v
3 h	7.98 ± 0.02	8.01 ± 0.01	8.01 ± 0.01	8.02 ± 0.02	8.01 ± 0.01
7 days	8.02 ± 0.01^a^	8.08 ± 0.01^b^	8.05 ± 0.01^ab^	8.04 ± 0.01^ab^	8.04 ± 0.01^ab^
14 days	8.03 ± 0.01^a^	8.04 ± 0.01^ab^	8.07 ± 0.02^ab^	8.07 ± 0.01^ab^	8.08 ± 0.01^b^
30 days	8.02 ± 0.01^a^	8.02 ± 0.02^a^	8.08 ± 0.01^b^	8.07 ± 0.01^ab^	8.05 ± 0.01^ab^
50 days	7.99 ± 0.01	8.00 ± 0.01	8.02 ± 0.01	8.01 ± 0.01	8.01 ± 0.01

## Discussion

### Effect of Digestate on Soil N Immobilisation and Availability

Digestate supplied soil with NH_4_-N, increasing concentrations of soil available nitrogen, which declined within the first week. Similarly, Alburquerque et al. [38] and Rigby and Smith [39] measured a rapid decrease in soil NH_4_-N after the initial input of NH_4_-N from the application of either liquid digestate or whole digestate. As NH_4_-N concentrations decreased, both studies measured a simultaneous increase in the nitrite and nitrate concentration from microbes oxidising NH_4_-N to obtain energy. This conversion of digestate supplied ammonium nitrogen into oxidised nitrogen kept soil total available nitrogen concentrations higher in digestate treated soils compared to soil only controls, which was also observed in our study. Yet unlike Alburquerque et al. [38] and Rigby and Smith [39], the magnitude of change in nitrite-nitrate concentrations in our study did not match the magnitude of change in NH_4_-N availability, and as there no significant difference in microbial nitrogen, this indicates that nitrogen losses occurred. As the pots did not contain drainage holes, no leaching could occur, therefore the losses were gaseous. Ammonia volatilisation is the cause for the greatest nitrogen losses shortly after application [5], but these losses had been expected to be low in this study due to incorporating the digestate immediately after application [40] and covering the pots loosely with lids to reduce airflow. Despite these measures, it is evident that greater volatilisation than anticipated occurred. Additionally, soil pH impacts volatilisation with greater fluxes observed in alkaline soil [41], such as the soil used in this study.

The addition of only digestate (without glycerol) in our study did not significantly stimulate microbial biomass production, compared to the soil only treatment, which is in agreement with de la Fuente et al., Wentzel and Joergensen and Valentinuzzi et al. [42–44] when they applied liquid digestate to soil. This can be attributed to the low amount of carbon supplied in the liquid fraction, which was 3.5% in the digestate used in this study. The low amount of carbon in the liquid fraction is a result of two steps. Firstly, during anaerobic digestion, the easily degradable carbon in the feedstock is utilised by microbes, decreasing the total carbon content of the digestate compared to its feedstock [45] and concurrently increasing the proportion of carbon which is recalcitrant [46] and harder for microbes to metabolise. Secondly post digestion separation, which is routinely done by biogas plants to reduce the volume of digestate for storage and transportation, removes a further 60–70% of the remaining carbon into the solid fraction [26], therefore the liquid digestate fraction contains low amounts of carbon for soil microbes to utilise.

*Effect of glycerol amended digestate on soil N immobilisation and availability.*

The addition of glycerol to the digestate resulted in lower soil available nitrogen concentrations than the digestate control from 7 days onwards. The difference observed at 7 days after application corresponded with higher microbial biomass, with the highest microbial biomass and lowest soil available nitrogen concentrations resulting from the 6% v/v and 9% v/v glycerol rates. This supports the first hypothesis that adding glycerol to digestate would increase microbial biomass, and nitrogen immobilisation therein. Yet the 9% v/v addition had no greater effect on microbial growth than 6% v/v. This indicates that beyond 6% v/v glycerol addition, other nutrients may have become a limiting factor for microbial growth.

Our study is in agreement with other lab-based studies that showed increases in microbial N when glycerol and nitrogen were co-applied compared to nitrogen fertilisers alone [21, 22, 47]. However, unlike Redmile-Gordon et al. and De, Sawyer and McDaniel [21, 22] who observed nitrogen remineralisation after 4 and 7 days of incubation respectively, there was no noticeable N mineralisation after the microbial N peak. This may have been due to unmeasured nitrogen losses shortly after application, as the addition of liquid digestate creates anaerobic microsites in the soil in which denitrifying activity takes place, which is further stimulated by the addition of labile carbon [48]. Therefore, the addition of glycerol could have led to an early loss of digestate supplied nitrogen as N_2_O and N_2_. Soil moisture returned to pre-application levels of 40% WHC after 7 days, which may have inhibited the rate of nitrogen mineralisation from organic matter due to low soil moisture conditions [49]. Yet it should be noted that this experiment did not contain a plant, which play a key role in stimulating nitrogen remineralisation through the secretion of rhizodeposits [50].

### Effect of Digestate and Glycerol on Soil Community Structure

The addition of only digestate to soil (without glycerol) resulted in a change to soil community structure, but no effect on F: B or G+:G- ratios were observed. The changes may be due to a combination of factors such as the microorganisms added by the digestate [51] and elevated soil pH, as changes in soil pH from by organic amendments applications have been found influence the microbial community composition [52]. The lack of change in F: B ratio was also observed by García-Sánchez et al. and Cattin et al. [53, 54], however Pezzolla et al. [55] observed an increase in Gram-negative bacteria which drove a reduction in the F: B ratio. This difference may be due to the feedstock of the digestate, as Pezzolla et al. [55] used digestate made from pig slurry, whereas the digestate used in our study came from a feedstock of plant material and manure, similar to the digestates used by García-Sánchez et al. and Cattin et al. [53, 54]. The carbon in digestates derived from pig slurry has a higher concentration of aliphatic forms, and is therefore more labile, compared to digestates derived from a feedstock mixture of plant and animal origin [46]. Gram-negative bacteria are better able to quickly utilise labile carbon due to their rapid growth strategy when there is a resource flush, compared to the relatively slower growing Gram-positive bacteria and slower growing fungi group [56]. However, assigning PLFA biomarkers to a specific microbial group should be done with caution, since some biomarkers are found across a range of organisms [57] and therefore changes in any one of these groups may be harder to compare between studies when different biomarkers are assigned to a particular group.

The addition of glycerol to digestate at 6 and 9% v/v resulted in a microbial group distinctly separate from the digestate alone from 14 days post-application onwards, supporting our second hypothesis. At both 14 and 50 days after application, glycerol addition at either 6 or 9% v/v did significantly reduce the G+:G- ratio. It is likely that this reduction can be attributed to positive effects on Gram-negative bacteria growth from labile C inputs, considering the positive effects measured on microbial biomass at the same time. This finding is supported by Garcia-Pausas and Paterson [58] and Cui et al. [59] who measured Gram-negative bacteria taking the majority of labile carbon (supplied as glucose in these studies), compared to other microbial groups. The increase of Gram-negative bacteria is likely the reason for the reduction in F:B ratio at day 50 under 9% v/v glycerol addition. These observations have important consequences for the fate of nitrogen in the soil organic matter. As Gram-negative bacteria are usually associated with quick growing and short-lived life strategies [60], with a rapid turnover of living biomass into the soil microbial food web and non-living soil organic matter [12], their necromass would increase the soil organic nitrogen pool more rapidly compared to the turnover from relatively slower growing Gram-positive bacteria and fungi. This in turn would ensure that nitrogen is available for plants to mineralise from the soil organic matter during their growing season.

### Further Considerations

There are several further steps that need to be taken to evaluate the practicality of digestate and glycerol co-application and to validate that this mechanism of using soil microbes to temporally immobilise digestate supplied nitrogen works in non-laboratory conditions. Firstly, to be effective as a fertiliser the nitrogen needs to remineralise whilst the crop is growing. As nitrogen remineralisation was not seen in this study, no assumptions can be made on the timing of nitrogen release. Two factors are proposed for the lack of remineralisation. Firstly, no plants were included in the incubation to prime soil organic matter decomposition, as such including plants to determine whether the addition of carbon additives to digestate does improve plant growth, nitrogen use efficiency and yield needs to be explored. Secondly no gaseous measurements were taken, which may have been a considerable nitrogen loss pathway. The influence of glycerol addition on N_2_O emissions is a major concern, as it is a potent greenhouse gas, as such quantifying the effect of glycerol addition to digestate on N_2_O emissions is important to determine the environmental sustainability of co-applying digestate and glycerol. Further studies using stable isotope probing are recommended to more accurately determine the pathways of digestate supplied nitrogen utilisation into the soil and its rate of transfer into the various nitrogen pools, and subsequent uptake in plants. Lastly the effects of glycerol addition on the soil community are unknown, although a start on understanding glycerol addition on soil microbial community is made in this experiment.Further work using DNA sequencing to determine the effect of glycerol addition on taxonomically distinct groups or functional gene expressions would contribute to a greater understanding of how the microbial community composition and functioning is affected.

## Conclusion

There is a growing body of research into the applicability of using labile carbon to immobilise nitrogen in the microbial biomass, and thereby reduce pollution from nitrogen rich sources. Our study contributes to this knowledge base by demonstrating for the first time that the addition of glycerol immobilises a significant proportion of the ammonium nitrogen supplied by anaerobic digestate into microbial biomass. This laboratory experiment was a proof of concept demonstrating the potential for both the biogas and biofuel industries to increase the value of their respective by-products by co-applying digestate and glycerol to soil to retain digestate supplied nitrogen in the soil, thereby mitigating the negative environmental impacts of applying digestate alone. Further experiments are necessary to evaluate the impacts on crop nitrogen use efficiency and yield, greenhouse gas emissions, and nitrate leaching. Although we have shown that glycerol addition stimulates the immobilisation of digestate nitrogen through its utilisation as a carbon source for microbial growth and biomass production, microorganisms also use glycerol to produce extracellular products, such as antibiotics [61] and extracellular polymeric substances [62]. Therefore, glycerol amendments could have broader applications for soil health, including improvements in soil structure and the potential for probiotic interventions to influence nutrient transformation and pathogen loads. Therefore, future studies should also investigate the effects of glycerol beyond its role as a growth substrate, focusing on microbial community composition and functioning. Additionally, field experiments will be necessary to understand how these parameters are affected by real-world conditions and to determine the practical viability for farmers to co-apply digestate and glycerol.

## Data Availability

The datasets generated during this study are available in the Cranfield University research data (CORD) repositoryat https://doi.org/10.57996/cran.ceres-2693.

## Declarations

### Statement of Novelty

The novelty of this study is its investigation into the effects of adding different rates of glycerol to the liquid fraction of anaerobic digestate on microbial biomass immobilisation of digestate supplied nitrogen. Furthermore, it explores the influence of glycerol addition of the microbial community through PLFA analysis including treatment impacts on both fungi: bacteria ratio and Gram-positive bacteria: Gram-negative bacteria ratio.
